# Quantifying walking speeds in relation to ankle biomechanics on a real-time interactive gait platform: a musculoskeletal modeling approach in healthy adults

**DOI:** 10.3389/fbioe.2024.1348977

**Published:** 2024-03-07

**Authors:** M. Peiffer, K. Duquesne, M. Delanghe, A. Van Oevelen, S. De Mits, E. Audenaert, A. Burssens

**Affiliations:** ^1^ Department of Orthopaedics and Traumatology, Ghent University Hospital, Ghent, Belgium; ^2^ Department of Human Structure and Repair, Ghent University, Ghent, Belgium; ^3^ Foot & Ankle Research and Innovation Lab (FARIL), Department of Orthopaedic Surgery, Massachusetts General Hospital, Harvard Medical School, Boston, MA, United States; ^4^ Department of Rheumatology, Ghent University Hospital, Ghent, Belgium; ^5^ Smart Space, Ghent University Hospital, Ghent, Belgium; ^6^ Department of Trauma and Orthopaedics, Addenbrooke’s Hospital, Cambridge University Hospitals NHS Foundation Trust, Cambridge, United Kingdom; ^7^ Department of Electromechanics, Op3Mech Research Group, University of Antwerp, Antwerp, Belgium

**Keywords:** gait-analysis, ankle joint, musculoskeletal modelling, computational biomechanics, walking speed

## Abstract

**Background:** Given the inherent variability in walking speeds encountered in day-to-day activities, understanding the corresponding alterations in ankle biomechanics would provide valuable clinical insights. Therefore, the objective of this study was to examine the influence of different walking speeds on biomechanical parameters, utilizing gait analysis and musculoskeletal modelling.

**Methods:** Twenty healthy volunteers without any lower limb medical history were included in this study. Treadmill-assisted gait-analysis with walking speeds of 0.8 m/s and 1.1 m/s was performed using the Gait Real-time Analysis Interactive Lab (GRAIL^®^). Collected kinematic data and ground reaction forces were processed via the AnyBody^®^ modeling system to determine ankle kinetics and muscle forces of the lower leg. Data were statistically analyzed using statistical parametric mapping to reveal both spatiotemporal and magnitude significant differences.

**Results:** Significant differences were found for both magnitude and spatiotemporal curves between 0.8 m/s and 1.1 m/s for the ankle flexion (*p* < 0.001), subtalar force (*p* < 0.001), ankle joint reaction force and muscles forces of the M. gastrocnemius, M. soleus and M. peroneus longus ( α = 0.05). No significant spatiotemporal differences were found between 0.8 m/s and 1.1 m/s for the M. tibialis anterior and posterior.

**Discussion:** A significant impact on ankle joint kinematics and kinetics was observed when comparing walking speeds of 0.8 m/s and 1.1 m/s. The findings of this study underscore the influence of walking speed on the biomechanics of the ankle. Such insights may provide a biomechanical rationale for several therapeutic and preventative strategies for ankle conditions.

## 1 Introduction

It is calculated that a human being undergoes approximately 6,000 steps a day. (Althoff et al., 2017; Paluch et al., 2022; Tison et al., 2022). Therefore, any discrepancy between the joint’s load-bearing capacity and the actual load it experiences often precipitates pathological changes within the ankle joint (Peiffer et al., 2023). Given the diversity in individual gait patterns, it is plausible that certain patterns may be associated with specific pathologies, such as ankle osteoarthrosis (Jarchi et al., 2018; Horst et al., 2021).

Gait analysis enables clinicians and researchers to investigate kinematic and kinetic parameters. This crucial biomechanical information can subsequently be used to establish diagnoses, evaluate therapeutic interventions, guide rehabilitation and more (van Dijsseldonk et al., 2018; Peri et al., 2019). However, current literature lacks comprehensive discussion on the influence of walking speed on the kinematics and kinetics of the ankle joint. It is a common approach to compare the gait biomechanics of pathological individuals to those of healthy individuals during gait analysis studies. However, it is essential to consider the influence of walking speed on an individual’s gait pattern, as pathological individuals often exhibit slower walking speeds compared to healthy adults. Booij et al. have previously shown that comparing total knee replacement patients with controls depends on the walking speed, and have provided a solution for speed correction using principal component analysis and full waveform analysis by use of statistical parametric mapping (Booij et al., 2021). Failing to account for this crucial factor can impede the validity and interpretability of the comparison (Fukuchi et al., 2019). Moreover, investigating pace in gait-analysis is not trivial. Several studies have previously investigated the influence of pace on ankle biomechanics, observing a higher range of motion, joint and muscle force in the ankle with increasing speed. Clinical protocols typically encompass walking distances ranging from 4 m to 10 m (Fineberg et al., 2013; van Hoeve et al., 2017; Schreiber and Moissenet, 2019; Klöpfer-Krämer et al., 2020; Alexander et al., 2021). However, the measurement of steady-state gait using these short tests presents several challenges in terms of standardization, as walking involves natural fluctuations in gait speed due to acceleration and deceleration. These factors can significantly impact the mean gait speed observed during such measurements. Treadmill-assisted gait analysis facilitates precise control and adjustment of the subject’s pace, presenting a potential solution for these inherent limitations. It is imperative to acknowledge that the locomotor patterns observed on the treadmill may exhibit constraints, as ambulation on a treadmill differs from overground walking. (Liu et al., 2016; Krumpoch et al., 2021).

**FIGURE 1:**
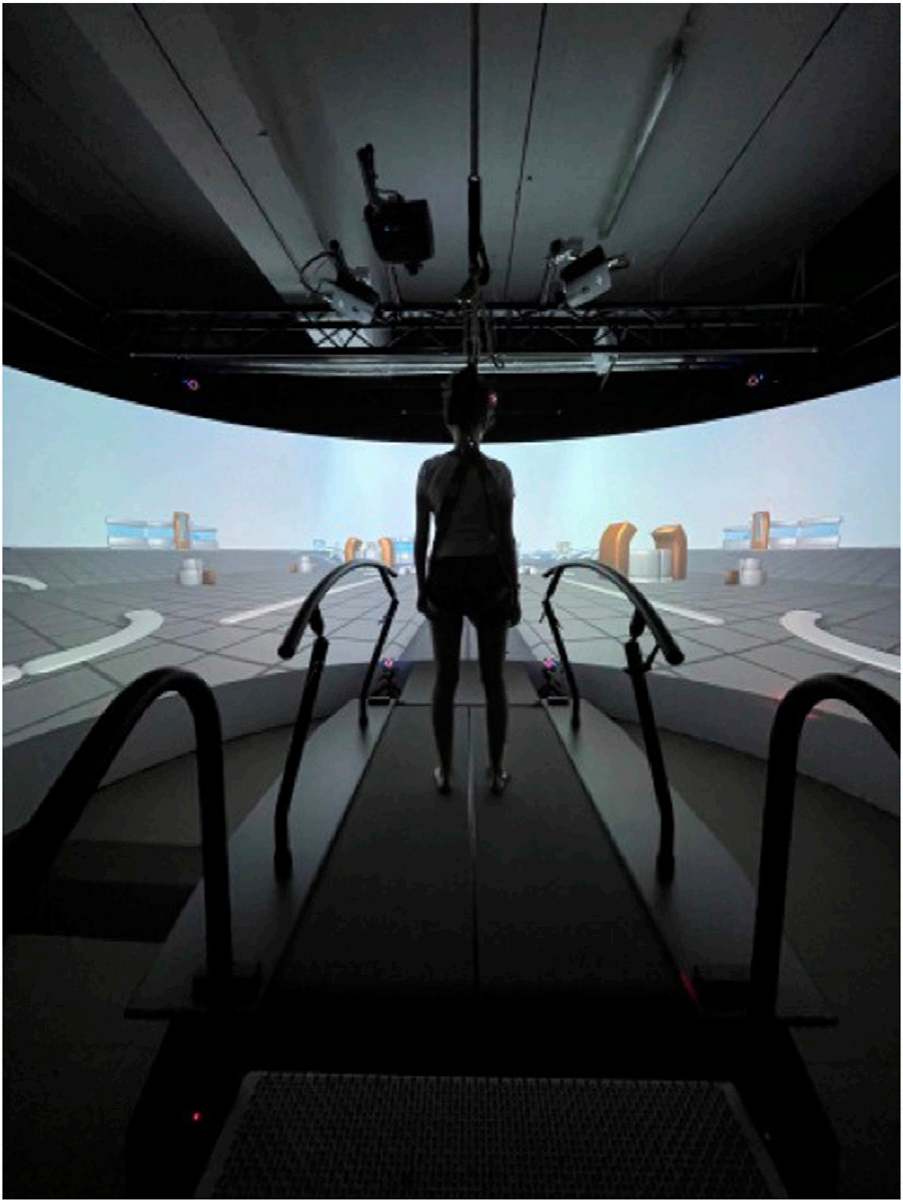
Shows the treadmill-assisted gait platform surrounded with screens to create a virtual reality experience to minimize the influence on the usual gait pattern.

The Gait Real-time analysis Interactive Lab (GRAIL, MotekForce Link Amsterdam BV, Netherlands) is a novel self-paced treadmill-assisted gait platform that incorporates a synchronized virtual reality environment on a semicircular screen (Figure 1). The instrument has found application in prior research endeavors; however, such applications have been circumscribed. The GRAIL remains distinctive as a platform uncommonly employed in the majority of medical centers. It takes the form of a treadmill-assisted gait platform encircled by screens, facilitating the creation of a virtual reality environment for the patient. It has been mostly used in previous studies focusing on balance training and motor control in patients with a history of stroke (de Rooij et al., 2021; Van Bladel et al., 2023), neuromuscular (Gagliardi et al., 2018; van Dijsseldonk et al., 2018) and chronic respiratory diseases (Liu et al., 2016). In case of age-related ankle problems, such as ankle arthritis, the GRAIL could stand out as an instrumental tool for in-depth exploration of the ankle’s biomechanical changes. Its integration into clinical practice has the potential to revolutionize treatment approaches by facilitating precise examinations and informed decision-making, ultimately improving the overall management of this age-related condition.

Advances in computational dynamics, such as those facilitated by the AnyBody Modeling System (Anybody Technology A/S, Aalborg, Denmark) (Damsgaard, 2006) or OpenSim (Delp et al., 2007; Seth et al., 2018), offer valuable tools for investigating internal forces and moments in the ankle joint, as well as muscle forces. By integrating anatomical data with motion capture information, it utilizes inverse dynamic optimization techniques to simulate the biomechanical behavior of the musculoskeletal system (Damsgaard, 2006; Van Houcke et al., 2020; Peiffer et al., 2022).

Therefore, this study aims to explore the potential impact of walking speed on ankle kinetics and kinematics using treadmill-assisted gait analysis. We will measure these variables at different pace, with the collected data subsequently analyzed via musculoskeletal modelling and simulations, where after statistical parametric mapping will be used to identify potential time-continuous differences (Liu et al., 2016; Motek, 2023). We hypothesize that higher walking speeds will lead to alterations in the ankle kinematics, joint reaction, and muscle forces on a real-time interactive gait platform.

## 2 Materials and methods

### 2.1 Study population

**FIGURE 2:**
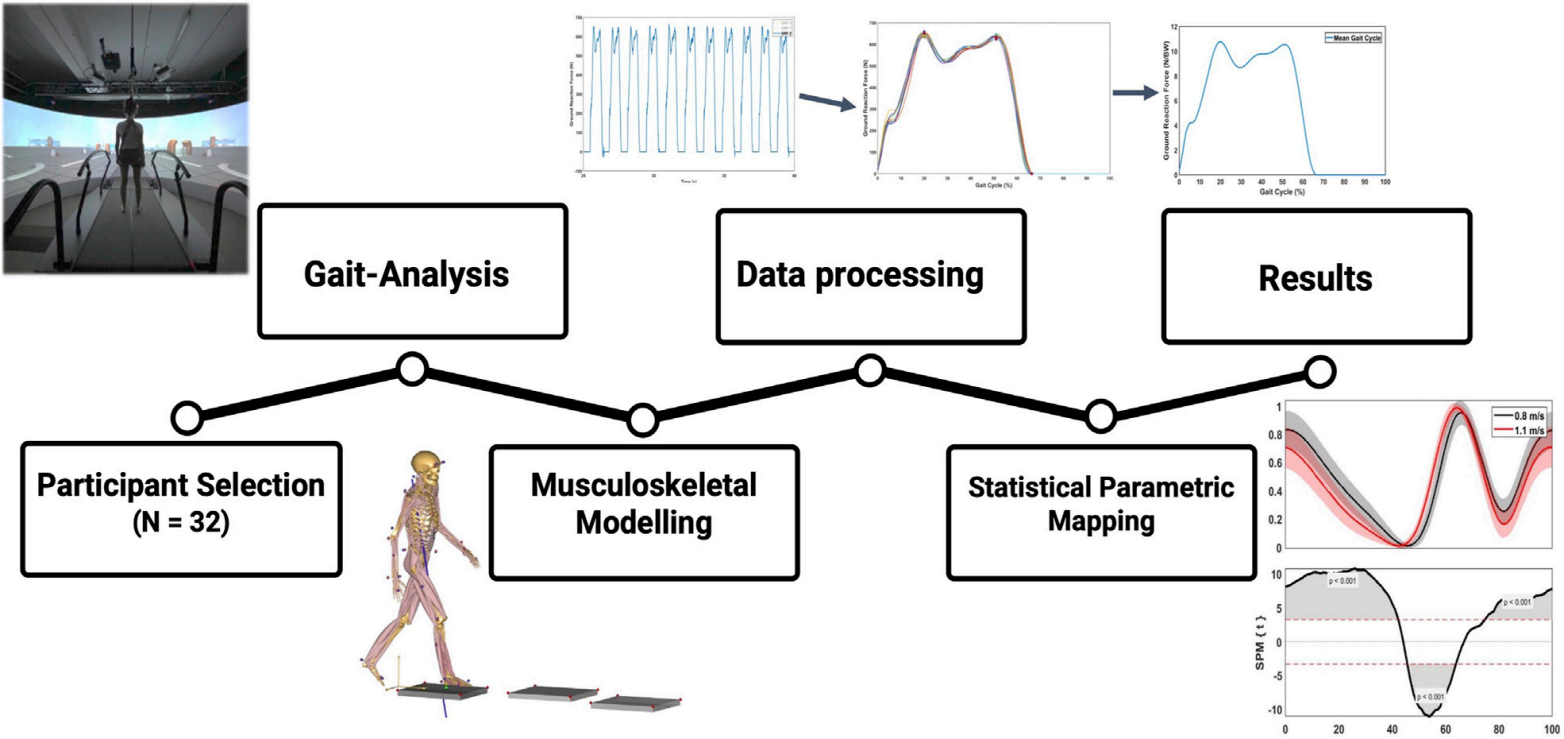
Methodology framework of this study. First, marker-based gait-analysis was performed in 20 young, healthy volunteers. Second, kinematics and GRF’s were transferred to AnyBody^®^ for musculoskeletal modelling and calculation of kinetic results. Third, raw results were processed in Matlab^®^ to perform time normalization and statistical parametric mapping.

A total of twenty healthy subjects volunteered to participate in this study. Demographic characteristics of our study population are listed in Table 1. Inclusion criteria consisted of an age between eighteen and 50 years old and being in a healthy and active condition without pre-existing ankle-, knee- or hip pathology or surgery during their lifetime. Exclusion criteria consisted of any medical history that could interfere with gait patterns and a musculoskeletal visual analogue pain rating scale higher than three at the moment of investigation (Karcioglu et al., 2018). The study was conducted in accordance with the Declaration of Helsinki and the Guidelines for Good Clinical Practice. The Institutional Review Board approved this study (IRB B6702021000905). Written consent was obtained from each subject prior to testing. The methodological framework of this study is presented in Figure 2.

**TABLE 1:** Demographic characteristics of the study population.

Age (yrs), mean (range) +- SD	28,75 +- 11,35 (19–50)
Gender distribution	12 females/8 males
Height (m), mean (range) +- SD	1,73 +- 0,11 (1,56–1,93)
Weight (kg), mean (range) +- SD	66,10 +- 9,75 (50–82)
BMI (kg/m^2^), mean	21,44 +- 2,13 (17,51–28,60)

### 2.2 Gait-analysis protocol

**FIGURE 3:**
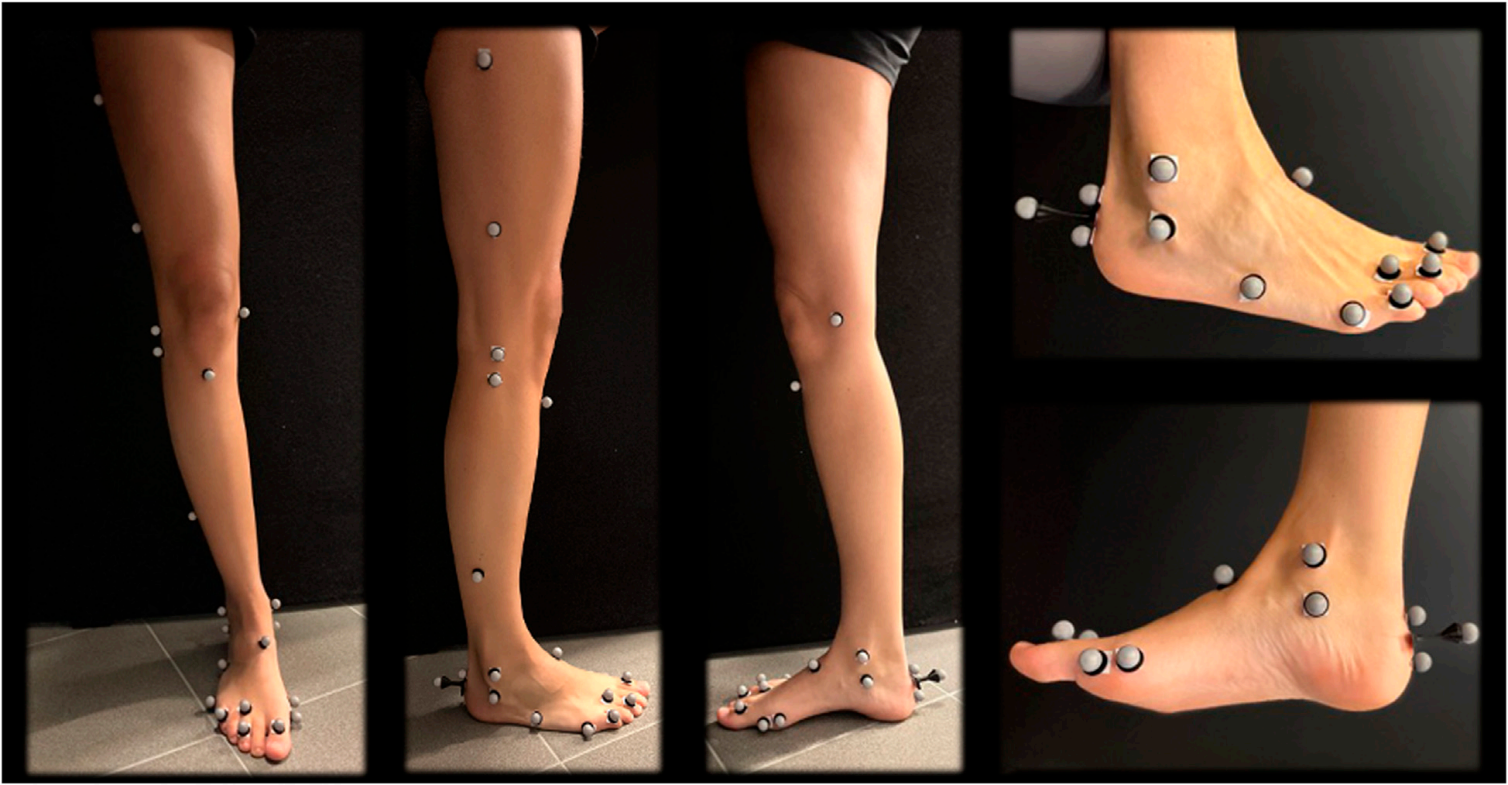
Marker protocol of the lower limb.

A total of 56 retro-reflective markers were stuck on the skin of the lower limbs on palpable landmarks. The marker protocol was based on a previous study by Kim et al. (Kim et al., 2018), which combined the plug-in-gait marker set and Oxford foot marker set along with three additional toe markers as seen in Figure 3 (Kadaba et al., 1989; Stebbins et al., 2006).

**FIGURE 4:**
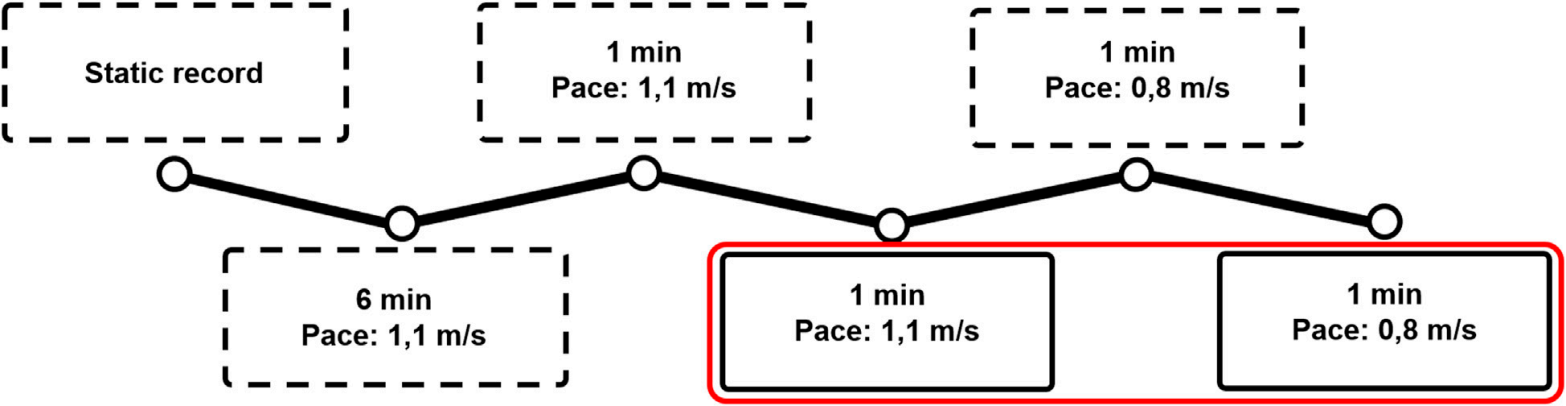
The protocol used for the gait analysis: First, a static trial and a 6-min familiarization walking trial at 1.1 m/s were performed. After that, the participant walked 1 min at each speed without analysis to get familiarized to the speed. First, 60 gait cycles at 1.1 m/s were collected, after which 60 cycles at 0.8 m/s were collected. Dotted frames represent non-collected trials, whereas the red frame represent the collected trials.

Motion capture was performed using the treadmill-assisted GRAIL (MotekForce Link Amsterdam BV, Netherlands). A virtual environment was projected on the 180° semicircular screen, involving a straight, endless path with industrial components on the side as depicted in Figure 1. In Figure 4, a flowchart of the gait-analysis protocol is presented. First, a static calibration record was performed, which comprised the participant standing upright with lower and upper limbs outstretched, palms facing forward, and a straight head. Subsequently, a 6-min familiarization walking trial at 1.1 m/s was performed. Collected gait-analysis consisted of 60 s at 1.1 m/s, followed by 60 s of slow walking at 0.8 m/s. Before each new pace, 1 minute of non-collected gait-analysis was performed for the participant to get used to the new pace (familiarization). Kinematic and Ground Reaction Force (GRF) data were saved and exported as. c3d files. It is demonstrated that healthy adults normally choose to walk at about 1.3 m s^−1^ (Bohannon, 1997). We selected a walking speed of 0.8 m/s, because this could also serve as a baseline reference in a patient cohort, as it demonstrated that patients with age related diseases like ankle osteoarthritis have an average walking speed of 0.8 m s^−1^ (Ingrosso et al., 2008). Additionally, we opted for 1.1 m/s as it is slightly below the mean walking speed, acknowledging that individuals tend to walk more slowly on a treadmill compared to overground walking and being able to have a faster control speed to compare patients in rehabilitation (being able to increase their walking speed on the treadmill but not yet to the extent of 1.3 m/s) (Song et al., 2020). The choice of outcome measures is based on their established relevance in previous examinations of ankle biomechanics (Riley et al., 2001; Liu et al., 2006; Fukuchi et al., 2019; Alexander et al., 2021).

### 2.3 Musculoskeletal modelling

Kinematic and GRF data were imported into the Anybody Modelling System (AMS version 7.1.0, Anybody Technology, Aalborg, Denmark). The Twente Lower Extremity Model (TLEM 2.0) which includes a two-segment foot model (modelling the ankle and subtalar joints separately), was scaled to each participant’s size using the length-mass-fat law proposed by Rasmussen and others (Rasmussen et al., 2005). The ankle and subtalar joint were modeled as a revolute joint with one rotational degree of freedom to allow flexion/extension and inversion/eversion motion, respectively. Joint kinematics were optimized by minimizing the differences between the experimental markers (captured by the cameras mounted on the GRAIL system) and the corresponding virtual markers on the models. Kinetics were calculated by using an inverse dynamics-based algorithm, implemented in the AnyBody Modelling System. Joint reaction forces (JRF) and joint moments were calculated at the rotation center of the respective joint. Muscle forces were scaled by use of the length-mass-fat scaling law and predicted to balance the external forces using the quadratic muscle recruitment criterion, as described more in depth in previous studies (Rasmussen et al., 2002; Damsgaard, 2006).

### 2.4 Data processing and statistical analysis

#### 2.4.1 Time normalization

**FIGURE 5:**
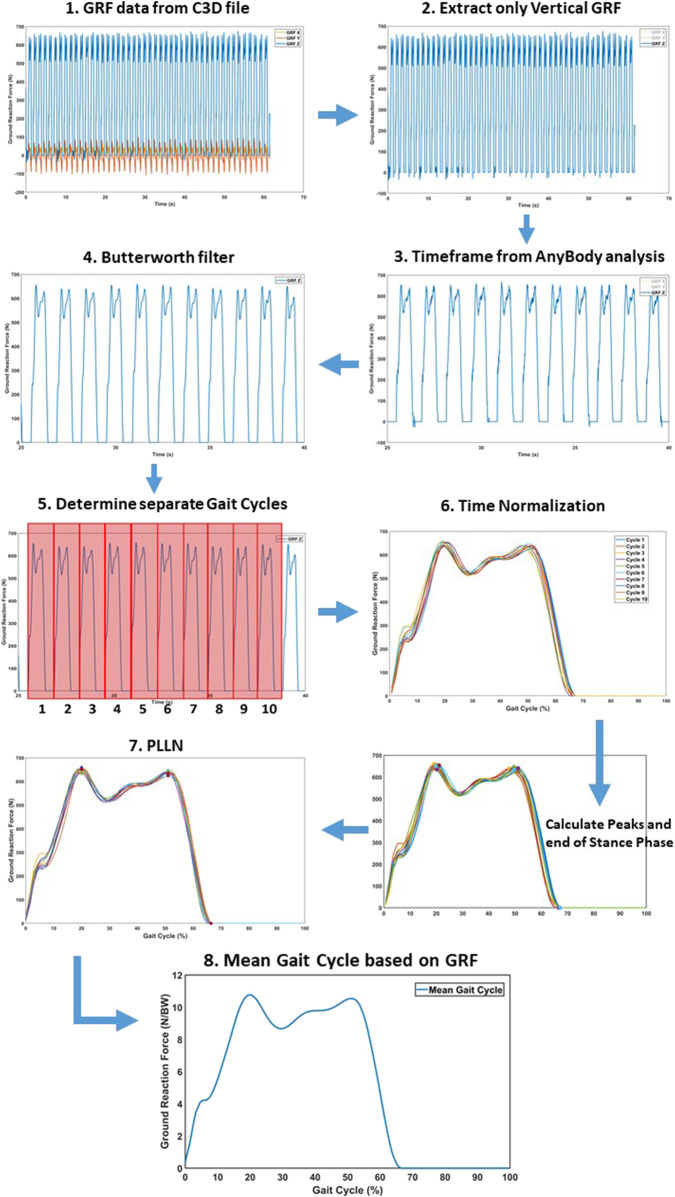
Time normalization protocol to convert the raw results into a separate mean gait cycle for statistical analysis. (1) Raw GRF data for the respective time frame. (2) Extracting only vertical GRF for alignment. (3) Matching the subset of the GRF to Anybody timeframe. (4) Butterworth filter to remove data noise. (5) Determine separate gate cycles by identifying the heel strike. (6) The different cycles were preliminary aligned, based on heel strike. (7) Piecewise Linear Length Normalization (PLLN) was performed, based on the end of the stance phase and two consistent GRF peaks. (8) The mean gait cycle, after PLLN.

Kinematic and kinetic data were transferred to a custom-made Matlab^®^ (Mathworks, Natick, MA, USA) script for further processing. Muscle forces and JRF’s were normalized to bodyweight (BW), while moments were normalized by the mass (kg). To remove noise, data were filtered using a sixth lowpass digital Butterworth filter with a normalized cutoff frequency of 12 Hz. Subsequently, a mean single gait cycle was obtained for each pace by averaging all gait cycles in a 25 s timeframe. The separate gait cycles contained within the 25s continuous recordings were separated and subsequently temporally aligned, upon heel strike detection. Next, a Piecewise Linear Length Normalization (PLLN) was performed to further align and normalize the separate gait cycles, similar to the previous study by Helwig et al. (2011). PLLN was automatically performed by computational identification of three consistent landmarks: the two consistent prominent peaks of the GRF curve (i.e., ‘maximum weight acceptance’ and ‘push-off’, respectively) and toe-off. After aligning all separate gait cycles, the mean gait cycle was achieved by averaging these separate cycles. All variables were aligned based on the GRF, after which the mean gait cycle curve for each variable was attained. This time normalization protocol is presented in Figure 5.

#### 2.4.2 Statistical parametric mapping

In order to investigate the time-continuous difference between the different paces, rather than a discrete analysis, Statistical Parametric Mapping (SPM) was performed for each variable by use of the Matlab ‘spm1d’ package (Pataky, 2010). SPM allowed to calculate statistically significant differences at each time point between different curves, taking into account the rest of the curve to calculate a statistically significant cutoff (Honert and Pataky, 2021). SPM has been most commonly used in functional magnetic resonance imaging as neuroimaging, but recent studies have successfully explored SPM also in gait analysis (Nieuwenhuys et al., 2017; Honert and Pataky, 2021; Alhossary et al., 2023). A 2-tailed SPM paired t-test compared the subject-averaged curves for each gait between two walking speeds (0.8 vs. 1.1 m/s). An alignment in time was performed to investigate the differences in magnitude, while a magnitude normalization (based on scaling the most prominent peak of the curves) was created to investigate spatiotemporal variations (Hu et al., 2005; Nieuwenhuys et al., 2017; Honert and Pataky, 2021).

## 3 Results

### 3.1 Ankle kinematics

#### 3.1.1 Ankle flexion

**FIGURE 6:**
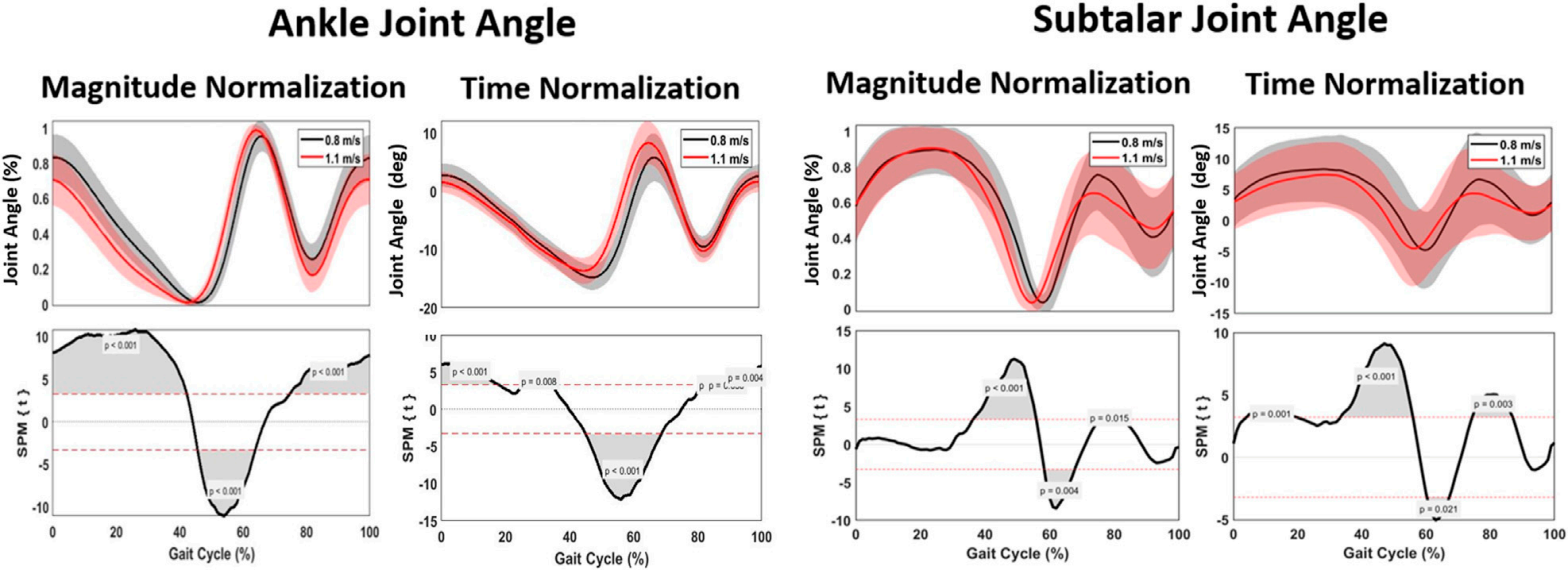
Mean gait cycles for ankle kinematics, regarding ankle joint angle and subtalar joint angle, comparing 0.8 m/s against 1.1 m/s pace.

A significant spatiotemporal difference of the ankle flexion curve between 0.8 m/s and 1.1 m/s was found for the whole gait cycle (*p* < 0.001), with the ankle plantar flexion occurring sooner at 0%–60% of the gait phase and ankle dorsiflexion occurring later at 75%–100% of the gait phase at 1.1 m/s. For magnitude, a significant difference was found between 0% and 10% representing greater plantarflexion at higher speed and 45%–65% representing greater dorsiflexion at higher speed of the gait cycle (Figure 6). A maximum ankle dorsiflexion of 15.01° was found for 0.8 m/s, in contrast to 13.91° for 1.1 m/s. At a pace of 1.1 m/s, the maximum angle of plantar flexion reached 8.35°, in contrast to 6.69° at 0.8 m/s (Table 2). With increasing velocity, a decrease in dorsiflexion angle was found, while the plantar flexion angle increased.

**TABLE 2:** Kinematic parameters. Statistically noted the maximum parameters for 0.8 m/s and 1.1 m/s and the significant magnitude or timing difference during the gait cycle.

Kinematic parameters	0.8 m/s (SD)	1.1 m/s (SD)	Significant magnitude difference (% of gait cycle, *p* < 0.05)	Significant timing difference (% of gait cycle, *p* < 0.05)
Maximum ankle plantar flexion (°)	6.69 (3.31)	8.58 (3.67)	0–15/85–100	0–40/70–100
Maximum ankle dorsiflexion (°)	15.01 (2.06)	13.91 (2.11)	45–70	45–65
Maximum subtalar inversion (°)	10.18 (6.92)	9.01 (6.75)	5–15/75–85	76–82
Maximum subtalar eversion (°)	5.39 (5.96)	5.07 (5.79)	35–60	37–57/60–65

#### 3.1.2 Subtalar version

Spatiotemporally, a significant difference between 40% and 65% was found for subtalar version; the transition from subtalar eversion to inversion occurred sooner when walking at 1.1 m/s. For magnitude, no difference was found for eversion, while a significant increase of 1.17° inversion was found for walking at 0.8 m/s (Figure 6).

### 3.2 Ankle kinetics

#### 3.2.1 External joint moments

**FIGURE 7:**
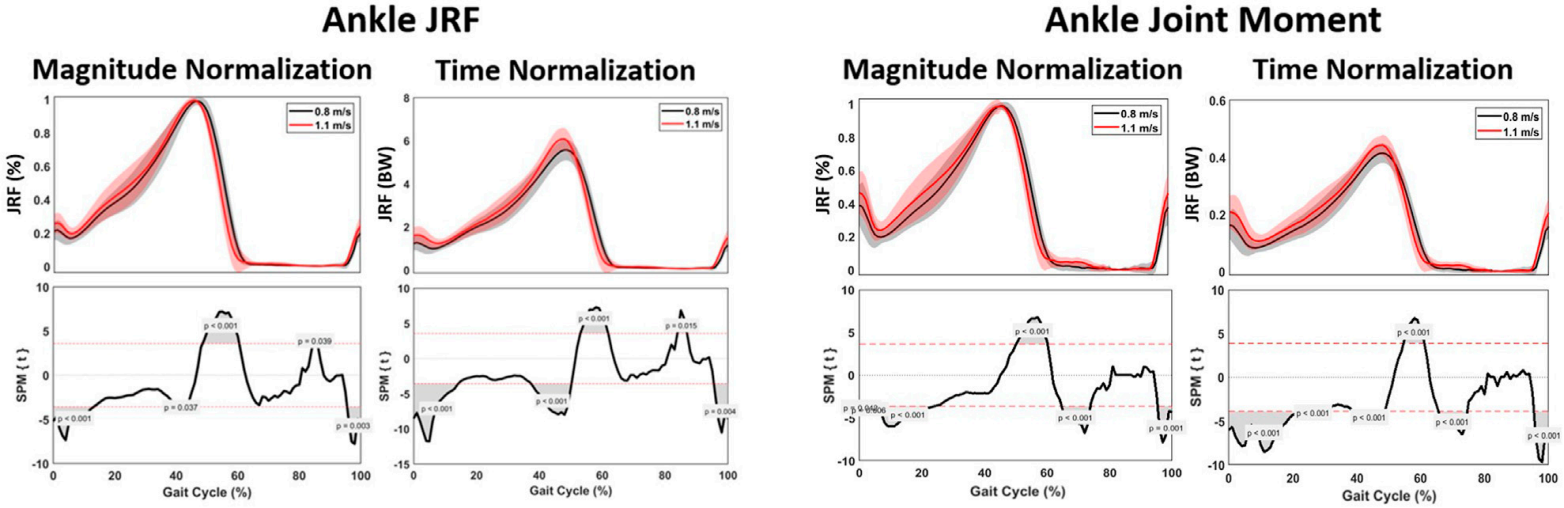
Mean gait cycles for muscle forces, including the M. soleus, M. gastrocnemius, M. tibialis posterior, M. tibialis anterior, M. peroneus longus and the M. peroneus brevis, comparing 0.8 m/s against 1.1 m/s pace.

The ankle joint moment showed a significant difference at 0%–20%, 55%–60%, 63%–73% and 90%–95% of the gait cycle, with the changes in ankle joint moment occurring sooner within the gait cycle at higher speed. There was a significant increase of 0.03 Nm/kg when walking at 1.1 m/s (Figure 7; Table 3).

**TABLE 3:** Kinetic parameters. Statistically noted the maximum parameters for 0.8 m/s and 1.1 m/s and the significant magnitude or timing difference during the gait cycle.

Kinetic parameters	0.8 m/s (SD)	1.1 m/s (SD)	Significant magnitude difference (% of gait cycle, *p* < 0.05)	Significant timing difference (% of gait cycle, *p* < 0.05)
Maximum ankle JRF (BW)	5.63 (0.45)	6.13 (0.53)	0–15/37–52/55–62/95–100	0–15/40–43/55–62/83–86/95–100
Maximum ankle JRF Moment (Nm/kg)	0.42 (0.03)	0.45 (0.04)	0–20/40–48/55–62/64–73/90–95	0–20/55–60/63–73/90–95

#### 3.2.2 Muscle forces

**FIGURE 8:**
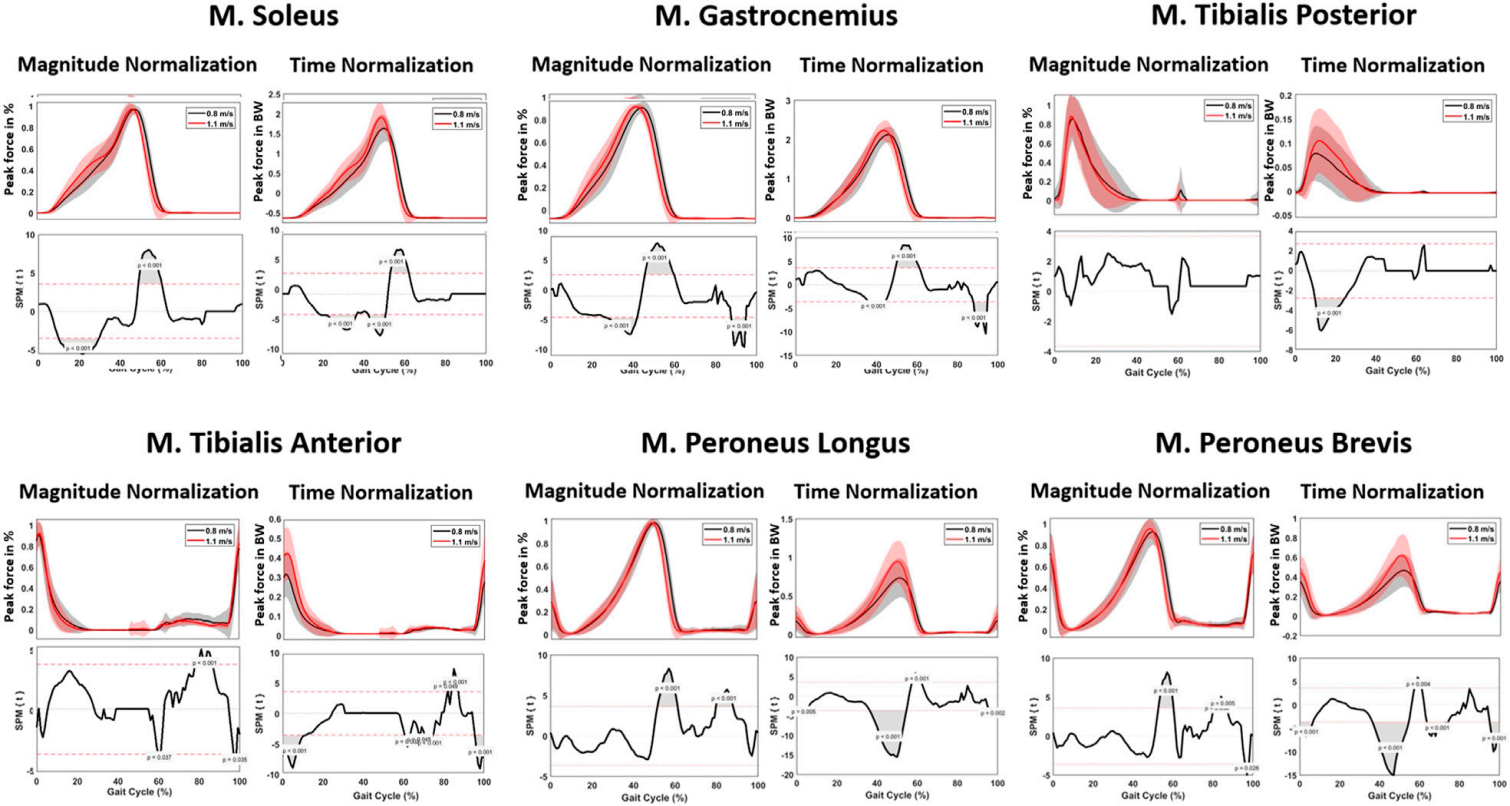
Kinetic parameters. Graphs comparing the ankle joint reaction force (left) and ankle joint moment (right) during the gait cycle between 0.8 m/s and 1.1 m/s.

When analyzing muscle forces involved in ankle and subtalar motion, changes in the required muscle force were observed. In the beginning of the gait cycle, the patterns were comparable across different walking speeds. However, starting from 45% of the gait cycle, a significant increase in muscle force development was found at 1.1 m/s for all muscle groups. Higher pace resulted in significantly faster attainment of peak force. Specifically, for the plantar flexors, the Musculus (M.) gastrocnemius and M. soleus, an increase of 0.09 and 0.21 times BW was observed at 1.1 m/s compared to 0.8 m/s (Figure 8; Table 4).

**TABLE 4:** Muscle parameters. Statistically noted the maximum parameters for 0.8 m/s and 1.1 m/s and the significant magnitude or timing difference during the gait cycle.

Muscle parameters (peak force in BW)	0.8 m/s (SD)	1.1 m/s (SD)	Significant magnitude difference (% of gait cycle, *p* < 0.05)	Significant timing difference (% of gait cycle, *p* < 0.05)
M. gastrocnemius	2.23 (0.20)	2.32 (0.27)	38–42/50–60/85–95	30–40/42–60/85–95
M. soleus	1.74 (0.22)	1.95 (0.21)	20–30/38–47/50–57	10–30/50–62
M. tibialis anterior	0.34 (0.12)	0.46 (0.13)	0–10/55–60/70–80	40–42/58–65/80–81
M. tibialis posterior	0.09 (0.06)	0.12 (0.07)	10–25	/
M. peroneus brevis	0.49 (0.15)	0.64 (0.21)	0–8/40–57/62–71	57–62/80–85
M. peroneus longus	0.75 (0.25)	0.96 (0.28)	40–60	52–62/80–87

#### 3.2.3 Joint reaction forces

Spatiotemporally, a significant difference at 0%–10%, 45%–60% and 95%–100% of the gait cycle was found for the ankle JRF, with the peak JRF occurring sooner within the gait cycle at higher speed. For magnitude, a significant increase of 0.5 times BW was observed when walking at 1.1 m/s, compared to 0.8 m/s. Furthermore, a significant lower ankle JRF was found during 0%–16%, 40%–50% and 95%–100% of the gait cycle (Figure 7).

## 4 Discussion

The objective of this study was to analyze the kinematics and kinetics of the ankle and subtalar joint in a group of healthy participants. Specifically, treadmill-assisted gait analysis was conducted using the GRAIL system at two different walking speeds of 0.8 m/s and 1.1 m/s. Joint angles, muscle forces, and joint reaction forces were calculated and compared across the different speeds. As hypothesized, the variations in walking speed had a significant impact on these parameters.

When investigating kinematics, an increase in plantarflexion and decrease in dorsiflexion was found when walking at 1.1 m/s compared to 0.8 m/s. Furthermore, a decrease in inversion was seen, while eversion remained constant. Plantarflexion occurred sooner in the gait cycle, corresponding to a faster attainment of terminal stance and pre-swing phase.

The analysis of kinetics revealed several differences, particularly in joint forces. Notably, a higher peak ankle JRF was found during midstance (i.e., the phase between heel strike and toe-off) at faster walking pace. More specifically, a mean peak force of 5.6 times BW was calculated at 0.8 m/s, compared to 6.1 times BW at 1.1 m/s. While this difference has not been shown in previous literature, the magnitude of these values are in agreement with previous studies (Brockett and Chapman, 2016; Prinold et al., 2016; Kim et al., 2018; Benemerito et al., 2020). These findings are consistent with previous studies by Dubbeldam et al. for the kinematic results and Riley et al. regarding the kinetic results (Riley et al., 2001; Dubbeldam et al., 2010). Alexander et al. have also found higher joint reaction forces for the ankle at 1.3 m/s than at 0.9 m/s, with corresponding values reported (approximately 6 times BW at 0.9 m/s and 6.3 times BW at 1.3 m/s) (Alexander et al., 2021). Additionally, our study showed that the peak joint reaction force occurred sooner in the gait cycle when walking at higher pace, presumably as a result of the peak plantarflexion occurring sooner in the gait cycle.

Regarding muscle forces, an increase in peak force of all muscle groups was found when walking at 1.1 m/s. This increase was most pronounced for the M. soleus (0.21 times BW) and M. peroneus longus (0.21 times BW). A similar trend was found in the literature, exhibiting greater muscle forces in faster walking speeds (Liu et al., 2006). Furthermore, Liu et al. found similar results for the muscles of the upper leg; namely, higher speed resulting in greater muscle forces (Liu et al., 2006). The findings of this study highlight the distinct muscle activation patterns associated with different gait phases. During the support phase, characterized by the initial heel strike, the M. tibialis anterior demonstrated significant activity, signifying its role in foot dorsiflexion. In the midstance phase, the M. tibialis posterior showed predominant activation, indicating its involvement in foot inversion. As the gait transitioned from midstance to propulsion, the M. gastrocnemius exhibited early activation, followed by pronounced engagement during the propulsion phase, jointly with the M. soleus, which played a crucial role in plantar flexion. In the transition from propulsion to the swing phase, the M. peroneus longus displayed notable activity, contributing to toe-off. Toward the end of the swing phase, both the dorsiflexors and eversion muscles demonstrated coordinated activation in preparation for the subsequent heel strike. These findings confirm the results of previous studies in this domain (Riley et al., 2001; Liu et al., 2006; Dubbeldam et al., 2010; Fukuchi et al., 2019).

The principal findings of our study, which revealed greater joint reaction forces and muscle forces acting on the ankle during higher walking pace, hold significant clinical implications that can enhance our understanding of human gait mechanics and have practical applications in clinical practice. For example, in patients with osteochondral lesions of the ankle, limiting the amount of joint reaction force causes less stress on the articular cartilage, and potentially less risk for additional mechanically-induced cartilage breakdown (Peiffer et al., 2023). Furthermore, knowledge of which muscles are most active during the specific gait phases allow clinicians and physiotherapists to target rehabilitation interventions to strengthen and stabilize the specific muscles at the appropriate time points within the gait cycle.

The foot and ankle are susceptible to age-related pathologies, such as ankle osteoarthritis, ankle instability and deformities (Barg et al., 2013; Peiffer et al., 2018; Burssens et al., 2022). These conditions induce alterations in ankle biomechanics, prompting a growing emphasis on exploring foot kinematics and gait analysis. The investigation of an individual’s biomechanics, specifically through a comprehensive gait analysis, holds substantial promise for these patients. Recognizing the nuanced variations in gait patterns among affected individuals can offer valuable insights into the progression and manifestation of these conditions (Valderrabano et al., 2007). The GRAIL system emerges as a possible optimal apparatus for the in-depth examination of such physiological dynamics. The utilization of GRAIL in clinical settings presents a promising avenue for advancing our understanding of these age-related diseases. Moreover, the implementation of the GRAIL system in clinical contexts could pave the way for the development of targeted therapeutic interventions. Since it can formulate precise and personalized treatment strategies, it holds the potential to enhance the overall quality of care for individuals grappling with these age-related afflictions.

The strengths of this study lie in the utilization of advanced technology such as the GRAIL system, assisted by treadmill, allowing for a continuous gait examination. Additionally, the AnyBody system was employed to estimate kinetics. The extensive use of markers on the foot and ankle allowed for a detailed examination of foot kinematics. Moreover, PLLN and SPM during statistical analysis made it possible to investigate both timing as magnitude significant differences during the whole gait cycle.

Several limitations of this study should be noted. First, as in all marked-based gait-analysis, errors in marker positioning can introduce errors in the described joint kinematics and subsequent calculation kinetics. By use of a multiple markers on the foot and ankle, this error was expected to be minimal. Furthermore, models were scaled using the length-mass-fat law, which is not as accurate as subject specific modelling (derived from medical imaging). Second, only young healthy participants in the age range of 18–50 years were included, without orthopedic or neurological conditions affecting gait. These do not represent the aging population. While this ensures reference values to be compared with further research in a pathological study group, it may not fully represent the aging population. Third, we have used a two-segment foot model, allowing for motion at the ankle and subtalar joint. Several previous studies have experimented with six-segment or even twenty-six-segment foot models, allowing for analysis of the different joints in the foot (Leardini et al., 1999; Forlani et al., 2015; Montefiori et al., 2022). An additional constraint necessitating consideration pertains to the sample size, which currently comprises only 20 subjects.

## 5 Conclusion

The findings of this study show that a higher walking pace significantly increases the peak joint reaction force and muscle force of the ankle. Furthermore, kinematic, and kinetic parameters exhibit timing differences between 0.8 m/s and 1.1 m/s walking pace. These results within young, healthy subjects may hold clinical implications for patients with foot and ankle conditions, such as rehabilitation choices to limit the forces exerted on the ankle joint. In research, it is vital to utilize standardized protocols that include predetermined walking speeds, enabling a reliable comparison of patients with average normative values.

## Data Availability

The data that support the findings of this study are not openly available due to reasons of sensitivity and are available from the corresponding author upon reasonable request.

## References

[B1] Alexander N. Schwameder H. Baker R. Trinler U. (2021). Effect of different walking speeds on joint and muscle force estimation using AnyBody and OpenSim. Gait Posture 90, 197–203. 10.1016/j.gaitpost.2021.08.026 34509042

[B2] Alhossary A. Pataky T. Ang W. T. Chua K. S. G. Kwong W. H. Donnelly C. J. (2023). Versatile clinical movement analysis using statistical parametric mapping in MovementRx. Sci. Rep. 13 (1), 2414. 10.1038/s41598-023-29635-4 36765193 PMC9918450

[B3] Althoff T. Sosič R. Hicks J. L. King A. C. Delp S. L. Leskovec J. (2017). Large-scale physical activity data reveal worldwide activity inequality. Nature 547 (7663), 336–339. 10.1038/nature23018 28693034 PMC5774986

[B4] Barg A. Pagenstert G. I. Hugle T. Gloyer M. Wiewiorski M. Henninger H. B. (2013). Ankle osteoarthritis: etiology, diagnostics, and classification. Foot Ankle Clin. 18 (3), 411–426. 10.1016/j.fcl.2013.06.001 24008208

[B5] Benemerito I. Modenese L. Montefiori E. Mazzà C. Viceconti M. Lacroix D. (2020). An extended discrete element method for the estimation of contact pressure at the ankle joint during stance phase. Proc. Inst. Mech. Eng. 234 (5), 507–516. 10.1177/0954411920905434 PMC7469707 32036769

[B6] Bohannon R. W. (1997). Comfortable and maximum walking speed of adults aged 20-79 years: reference values and determinants. Age Ageing 26 (1), 15–19. 10.1093/ageing/26.1.15 9143432

[B7] Booij M. J. Meinders E. Sierevelt I. N. Nolte P. A. Harlaar J. van den Noort J. C. (2021). Matching walking speed of controls affects identification of gait deviations in patients with a total knee replacement. Clin. Biomech. 82, 105278. 10.1016/j.clinbiomech.2021.105278 33540284

[B8] Brockett C. L. Chapman G. J. (2016). Biomechanics of the ankle. Orthop. Trauma 30 (3), 232–238. 10.1016/j.mporth.2016.04.015 27594929 PMC4994968

[B9] Burssens A. Krähenbühl N. Lenz A. L. Howell K. Zhang C. Sripanich Y. (2022). Interaction of loading and ligament injuries in subtalar joint instability quantified by 3D weightbearing computed tomography. J. Orthop. Res. Off. Publ. Orthop. Res. Soc. 40 (4), 933–944. 10.1002/jor.25126 34191355

[B10] Damsgaard M. (2006). Analysis of musculoskeletal systems in the AnyBody modeling system. Amsterdam, Netherlands: Elsevier.

[B11] Delp S. L. Anderson F. C. Arnold A. S. Loan P. Habib A. John C. T. (2007). OpenSim: open-source software to create and analyze dynamic simulations of movement. IEEE Trans. Biomed. Eng. 54 (11), 1940–1950. 10.1109/tbme.2007.901024 18018689

[B12] de Rooij I. J. M. van de Port I. G. L. Punt M. Abbink-van Moorsel P. J. M. Kortsmit M. van Eijk R. P. A. (2021). Effect of virtual reality gait training on participation in survivors of subacute stroke: a randomized controlled trial. Phys. Ther. 101 (5), pzab051. 10.1093/ptj/pzab051 33594443 PMC8122468

[B13] Dubbeldam R. Buurke J. H. Simons C. Groothuis-Oudshoorn C. G. Baan H. Nene A. V. (2010). The effects of walking speed on forefoot, hindfoot and ankle joint motion. Clin. Biomech. Bristol Avon 25 (8), 796–801. 10.1016/j.clinbiomech.2010.06.007 20619515

[B15] Fineberg D. B. Asselin P. Harel N. Y. Agranova-Breyter I. Kornfeld S. D. Bauman W. A. (2013). Vertical ground reaction force-based analysis of powered exoskeleton-assisted walking in persons with motor-complete paraplegia. J. Spinal Cord. Med. 36 (4), 313–321. 10.1179/2045772313y.0000000126 23820147 PMC3758528

[B16] Forlani M. Sancisi N. Parenti-Castelli V. (2015). A three-dimensional ankle kinetostatic model to simulate loaded and unloaded joint motion. J. Biomech. Eng. 137, 061005. 10.1115/1.4029978 25751452

[B17] Fukuchi C. A. Fukuchi R. K. Duarte M. (2019). Effects of walking speed on gait biomechanics in healthy participants: a systematic review and meta-analysis. Syst. Rev. 8 (1), 153. 10.1186/s13643-019-1063-z 31248456 PMC6595586

[B18] Gagliardi C. Turconi A. C. Biffi E. Maghini C. Marelli A. Cesareo A. (2018). Immersive virtual reality to improve walking abilities in cerebral palsy: a pilot study. Ann. Biomed. Eng. 46 (9), 1376–1384. 10.1007/s10439-018-2039-1 29704186

[B19] Helwig N. E. Hong S. Hsiao-Wecksler E. T. Polk J. D. (2011). Methods to temporally align gait cycle data. J. Biomech. 44 (3), 561–566. 10.1016/j.jbiomech.2010.09.015 20887992

[B20] Honert E. C. Pataky T. C. (2021). Timing of gait events affects whole trajectory analyses: a statistical parametric mapping sensitivity analysis of lower limb biomechanics. J. Biomech. 119, 110329. 10.1016/j.jbiomech.2021.110329 33652238

[B21] Horst F. Slijepcevic D. Simak M. Schöllhorn W. I. (2021). Gutenberg Gait Database, a ground reaction force database of level overground walking in healthy individuals. Sci. Data 8 (1), 232. 10.1038/s41597-021-01014-6 34475412 PMC8413275

[B22] Hu D. Yan L. Liu Y. Zhou Z. Friston K. J. Tan C. (2005). Unified SPM-ICA for fMRI analysis. NeuroImage 25 (3), 746–755. 10.1016/j.neuroimage.2004.12.031 15808976

[B23] Ingrosso S. Benedetti M. Leardini A. Casanelli S. Giannini S. (2008). Gait analysis of a novel design of ankle replacement. J. Foot Ankle Res. 1 (Suppl. 1), P1. 10.1186/1757-1146-1-s1-p1

[B24] Jarchi D. Pope J. Lee T. K. M. Tamjidi L. Mirzaei A. Sanei S. (2018). A review on accelerometry-based gait analysis and emerging clinical applications. IEEE Rev. Biomed. Eng. 11, 177–194. 10.1109/rbme.2018.2807182 29994786

[B25] Kadaba M. P. Ramakrishnan H. K. Wootten M. E. Gainey J. Gorton G. Cochran G. V. (1989). Repeatability of kinematic, kinetic, and electromyographic data in normal adult gait. J. Orthop. Res. Off. Publ. Orthop. Res. Soc. 7 (6), 849–860. 10.1002/jor.1100070611 2795325

[B26] Karcioglu O. Topacoglu H. Dikme O. Dikme O. (2018). A systematic review of the pain scales in adults: which to use? Am. J. Emerg. Med. 36 (4), 707–714. 10.1016/j.ajem.2018.01.008 29321111

[B27] Kim Y. Lee K. M. Koo S. (2018). Joint moments and contact forces in the foot during walking. J. Biomech. 74, 79–85. 10.1016/j.jbiomech.2018.04.022 29735264

[B28] Klöpfer-Krämer I. Brand A. Wackerle H. Müßig J. Kröger I. Augat P. (2020). Gait analysis – available platforms for outcome assessment. Injury 51 (Suppl. 2), S90–s96. 10.1016/j.injury.2019.11.011 31767371

[B29] Krumpoch S. Lindemann U. Rappl A. Becker C. Sieber C. C. Freiberger E. (2021). The effect of different test protocols and walking distances on gait speed in older persons. Aging Clin. Exp. Res. 33 (1), 141–146. 10.1007/s40520-020-01703-z 32930990 PMC7897617

[B30] Leardini A. O’Connor J. J. Catani F. Giannini S. (1999). A geometric model of the human ankle joint. J. Biomech. 32 (6), 585–591. 10.1016/s0021-9290(99)00022-6 10332622

[B31] Liu M. Q. Anderson F. C. Pandy M. G. Delp S. L. (2006). Muscles that support the body also modulate forward progression during walking. J. Biomech. 39 (14), 2623–2630. 10.1016/j.jbiomech.2005.08.017 16216251

[B32] Liu W. Y. Meijer K. Delbressine J. M. Willems P. J. Franssen F. M. Wouters E. F. (2016). Reproducibility and validity of the 6-minute walk test using the gait real-time analysis interactive lab in patients with COPD and healthy elderly. PloS One 11 (9), e0162444. 10.1371/journal.pone.0162444 27607426 PMC5015964

[B33] Montefiori E. Fiifi Hayford C. Mazzà C. (2022). Variations of lower-limb joint kinematics associated with the use of different ankle joint models. J. Biomech. 136, 111072. 10.1016/j.jbiomech.2022.111072 35397320

[B34] Motek (2023). Grail-The ultimate gate-lab solution. Available from: https://www.motekmedical.com/solution/grail/.

[B35] Nieuwenhuys A. Papageorgiou E. Desloovere K. Molenaers G. De Laet T. (2017). Statistical parametric mapping to identify differences between consensus-based joint patterns during gait in children with cerebral palsy. PloS One 12 (1), e0169834. 10.1371/journal.pone.0169834 28081229 PMC5231378

[B36] Paluch A. E. Bajpai S. Bassett D. R. Carnethon M. R. Ekelund U. Evenson K. R. (2022). Daily steps and all-cause mortality: a meta-analysis of 15 international cohorts. Lancet Public Health 7 (3), e219–e228. 10.1016/s2468-2667(21)00302-9 35247352 PMC9289978

[B37] Pataky T. C. (2010). Generalized n-dimensional biomechanical field analysis using statistical parametric mapping. J. Biomech. 43 (10), 1976–1982. 10.1016/j.jbiomech.2010.03.008 20434726

[B38] Peiffer M. Belvedere C. Clockaerts S. Leenders T. Leardini A. Audenaert E. (2018). Three-dimensional displacement after a medializing calcaneal osteotomy in relation to the osteotomy angle and hindfoot alignment. Foot Ankle Surg. 26, 78–84. 10.1016/j.fas.2018.11.015 30581061

[B39] Peiffer M. Burssens A. Duquesne K. Last M. De Mits S. Victor J. (2022). Personalised statistical modelling of soft tissue structures in the ankle. Comput. Methods Programs Biomed. 218, 106701. 10.1016/j.cmpb.2022.106701 35259673

[B40] Peiffer M. Duquesne K. Van Oevelen A. Burssens A. De Mits S. Maas S. A. (2023). Validation of a personalized ligament-constraining discrete element framework for computing ankle joint contact mechanics. Comput. Methods Programs Biomed. 231, 107366. 10.1016/j.cmpb.2023.107366 36720186

[B41] Peri E. Panzeri D. Beretta E. Reni G. Strazzer S. Biffi E. (2019). Motor improvement in adolescents affected by ataxia secondary to acquired brain injury: a pilot study. Biomed. Res. Int. 2019, 1–8. 10.1155/2019/8967138 PMC6899307 31886263

[B42] Prinold J. A. Mazzà C. Di Marco R. Hannah I. Malattia C. Magni-Manzoni S. (2016). A patient-specific foot model for the estimate of ankle joint forces in patients with juvenile idiopathic arthritis. Ann. Biomed. Eng. 44 (1), 247–257. 10.1007/s10439-015-1451-z 26374518 PMC4690839

[B43] Rasmussen J. Damsgaard M. Christensen S. T. Surma E. (2002). Design optimization with respect to ergonomic properties. Struct. Multidiscip. Optim. 24 (2), 89–97. 10.1007/s00158-002-0219-x

[B44] Rasmussen J. de Zee M. Damsgaard M. Tørholm S. Marek C. Siebertz K. (2005). A general method for scaling musculo-skeletal models. Denmark: Aalborg University.

[B45] Riley P. O. DellaCroce U. Kerrigan D. C. (2001). Effect of age on lower extremity joint moment contributions to gait speed. Gait Posture 14 (3), 264–270. 10.1016/s0966-6362(01)00133-3 11600330

[B46] Schreiber C. Moissenet F. (2019). A multimodal dataset of human gait at different walking speeds established on injury-free adult participants. Sci. Data 6 (1), 111. 10.1038/s41597-019-0124-4 31270327 PMC6610108

[B47] Seth A. Hicks J. L. Uchida T. K. Habib A. Dembia C. L. Dunne J. J. (2018). OpenSim: simulating musculoskeletal dynamics and neuromuscular control to study human and animal movement. PLoS Comput. Biol. 14 (7), e1006223. 10.1371/journal.pcbi.1006223 30048444 PMC6061994

[B48] Song S. Choi H. Collins S. H. (2020). Using force data to self-pace an instrumented treadmill and measure self-selected walking speed. J. NeuroEngineering Rehabil. 17, 68. 10.1186/s12984-020-00683-5 PMC7268460 32493426

[B49] Stebbins J. Harrington M. Thompson N. Zavatsky A. Theologis T. (2006). Repeatability of a model for measuring multi-segment foot kinematics in children. Gait Posture 23 (4), 401–410. 10.1016/j.gaitpost.2005.03.002 15914005

[B50] Tison G. H. Barrios J. Avram R. Kuhar P. Bostjancic B. Marcus G. M. (2022). Worldwide physical activity trends since COVID-19 onset. Lancet Glob. Health 10 (10), e1381–e1382. 10.1016/s2214-109x(22)00361-8 36057269 PMC9432866

[B51] Valderrabano V. Nigg B. M. von Tscharner V. Stefanyshyn D. J. Goepfert B. Hintermann B. (2007). Gait analysis in ankle osteoarthritis and total ankle replacement. Clin. Biomech. 22 (8), 894–904. 10.1016/j.clinbiomech.2007.05.003 17604886

[B52] Van Bladel A. De Ridder R. Palmans T. Van der Looven R. Cambier D. (2023). Comparing spatiotemporal gait parameters between overground walking and self-paced treadmill walking in persons after stroke. Disabil. Rehabil. 45 (6), 1016–1021. 10.1080/09638288.2022.2046875 35332811

[B53] van Dijsseldonk R. B. de Jong L. A. F. Groen B. E. Vos-van der Hulst M. Geurts A. C. H. Keijsers N. L. W. (2018). Gait stability training in a virtual environment improves gait and dynamic balance capacity in incomplete spinal cord injury patients. Front. Neurol. 9, 963. 10.3389/fneur.2018.00963 30524356 PMC6256239

[B54] van Hoeve S. Leenstra B. Willems P. Poeze M. Meijer K. (2017). The effect of age and speed on foot and ankle kinematics assessed using a 4-segment foot model. Med. Baltim. 96 (35), e7907. 10.1097/md.0000000000007907 PMC5585503 28858109

[B55] Van Houcke J. Galibarov P. E. Van Acker G. Fauconnier S. Allaert E. Van Hoof T. (2020). Personalized hip joint kinetics during deep squatting in young, athletic adults. Comput. Methods Biomech. Biomed. Engin 23 (1), 23–32. 10.1080/10255842.2019.1699539 31818133

